# Severity of Caregiver Stress in Relation to Severity of Disease in Persons with Parkinson’s

**DOI:** 10.7759/cureus.4358

**Published:** 2019-04-01

**Authors:** Haresh Kumar, Shahbaz Ansari, Vikash Kumar, Habiba D Barry, Amber Tahir

**Affiliations:** 1 Internal Medicine, United Medical and Dental College, Karachi, PAK; 2 Internal Medicine, Jinnah Postgraduate Medical Center, Karachi, PAK; 3 Internal Medicine, Jinnah Sindh Medical University, Karachi, PAK; 4 Internal Medicine, Dow University of Health Sciences, Karachi, PAK

**Keywords:** caregiver health, stress in caregiver, parkinson disease, hoehn and yahr scale, caregiver burden inventory

## Abstract

Introduction

Parkinson’s disease (PD) is a progressive neurological disorder. It presents with motor symptoms and gradually progresses to cognitive impairment. It has debilitating impact not only on the psychological health of the patient but also of the caregivers. The aim of this study is to evaluate the stress level among caregivers of PD and assess its correlation with the disease factors including duration, severity, and presence of on-off phenomenon.

Methods

One hundred and fifty-six patients of Parkinson’s disease and their caregivers were enrolled. Severity of Parkinson’s disease was assessed using Hoehn and Yahr Scale. Caregiver stress was evaluated using Caregiver Burden Inventory. Data was entered and analyzed using Statistical Package for the Social Sciences (SPSS) version 22.0 (IBM Corp., Armonk, NY, USA).

Results

There were 112 (71.8%) women and 44 (28.2%) men in the caregiver group. Their mean age was 47.75 ± 11.98 years. There were 98 (62.8%) stressed caregivers and 58 (37.2%) non-stressed caregivers. In patients with stage 4 and 5 PD, 67-80% caregivers were stressed as compared to only 28% caregivers of stage 1 PD (p-value = 0.0008). Duration of Parkinson’s disease more than 10 years and presence of on-off phenomenon was also significantly associated with higher stress in the caregivers (p-value < 0.00001; p-value = 0.002, respectively). Among the stressed caregivers, 85 (86.7%) were women and only 13 (13.3%) were men (p-value < 0.0001).

Conclusion

Psychological health of caregivers of persons with Parkinson’s is bleak. As the disease progresses, they further succumb to debilitating stress and depression. Qualitative and quantitative studies must be conducted in Pakistan to understand the psychosocial status of Parkinson’s disease caregivers and plan strategies to improve their quality of life.

## Introduction

Parkinson’s disease (PD) is an age-related, progressive, neurodegenerative disorder which commonly presents as resting tremor, rigidity, bradykinesia or slowness, gait disturbance, and postural instability. By 2016, Parkinson’s disease has affected 6.1 million people globally [1, 2].

Like all other chronic diseases, Parkinson’s also has negative psychosocial effects. These include depression, anxiety, social phobia, and panic disorder [3]. As the disease progresses, it also leads to the development of cognitive deficits and eventually dementia. With a mean age at onset of 55, and the markedly increasing incidence with age, societies are expected to face an increasing social and economic burden with time [4, 5]. The major percentage of this burden befalls the caregivers of patients with PD, who are challenged with emotional, financial, and physical trials on a day to day basis. These tasks often cause caregivers to neglect their own needs for sleep, nutrition, and health care and can result in caregiver burnout [6].

The discovery of levodopa revolutionized the treatment of Parkinson’s disease, which largely, comprises the current treatment strategy [1]. However, patients with Parkinson’s, on chronic levodopa therapy, may experience fluctuations (the "on-off" effect), which is a transition from "mobility to disability," i.e. "on to off" state, often several times a day [7]. With disease progression and onset of "on-off phenomenon," the physical dependence of the patient increases. Higher daily life physical dependency and increased motor symptoms have shown the highest predisposition to caregiver distress [8]. The process of caring for a physically dependent individual strains their mental and physical health, their social relationships, and their quality of life. The imminent realization and contemplation of a loved one’s death is also an additional stress trigger. In O'Reilly et al., spouses of patients with Parkinson’s reported depression and psychological stress more than healthy elderly controls. They suggested that mental disturbances were the most significant determinants of caregiver stress [9]. The clinicians need to pay close attention to the psychological health of caregivers of patients with Parkinson’s disease, particularly in spouses.

The aim of this study is to evaluate the stress level among caregivers of Parkinson’s disease and assess its correlation with the disease factors including duration, severity, and presence of on-off phenomenon.

## Materials and methods

Study setting and design

The study was carried out in the neurology department of Jinnah Postgraduate Medical Centre from June till December 2018. It was a prospective observational study. The department conducts Parkinson’s outpatient clinic once weekly. The sampling technique was consecutive and convenient.

Study participants

The study comprised of two groups of participants. “Patients” group were the persons diagnosed with PD who were being followed up in the weekly clinic. The inclusion criteria for “patients” group consisted of patients of both genders who had been diagnosed with PD for at least one year. For this group, duration of PD, presence of on-off phenomenon, and severity on Hoehn and Yahr Scale were recorded [10]. On-off phenomenon was taken as phases of mobility and immobility in PD patients being managed with levodopa. This switch of mobility to immobility and vice versa occurs due to “wearing-off” of the drug or as sudden motor fluctuations [7].

The second group of participants was “caregivers.” Caregivers were individuals of both genders who lived with at least one person with PD (diagnosed for more than one year); they could be a parent, sibling, spouse, relative, or even a friend [11]. Caregivers who moved in with persons with PD within less than one year were not included. The “caregivers” group completed a self-reported questionnaire which included their gender and the Caregiver Burden Inventory (CBI) [12].

All acts were conducted after complete understanding and written consent of both groups of participants. Ethical approval was obtained from the institutional review board.

Study instruments

*a*. *Hoehn and Yahr Scale (HY scale)*

HY scale is a clinical scale used to categorize patients of PD with respect to the severity of their motor symptoms. It captures progressive motor impairment characteristic in PD and its stages correspond to deteriorating motor function. It has five stages. Stage 1 is the least severe and is characterized by “Unilateral involvement only usually with minimal or no functional disability.” Stage 2 is characterized by “Bilateral or midline involvement without impairment of balance.” Stage 3 is characterized by “Bilateral disease: mild to moderate disability with impaired postural reflexes; physically independent.” Stage 4 is characterized by “Severely disabling disease; still able to walk or stand unassisted” and stage 5 is characterized by “Confinement to bed or wheelchair unless aided” [10].

*b*. *Caregiver Burden Inventory (CBI)*

CBI was developed in the 1980s and has been used widely since then. It is a multidimensional approach which evaluates the burden on caregiver on five parameters including time dependency, emotional health, physical health, development, and social relationships. It also provides a global score which masks the discrepancies between the extents of burden on individual parameter. A global CBI score of ≥36 indicates greater stress and need for respite. Internal consistency reliability (Coefficient Alpha) of factors 1 and 2 was 0.85 each. Factors 3, 4, and 5 had Alpha values of 0.86, 0.73, and 0.77, respectively [12].

Data analysis

All data were entered and analyzed using the Statistical Package for the Social Sciences (SPSS) version 22.0 (IBM Corp., Armonk, NY, USA) for Windows. Patients of PD were classified according to the HY scale. Caregivers with global CBI score ≥36 were termed as “stressed.” CBI scores of caregivers were stratified for their genders and three parameters of PD patients - severity, duration, and on-off phenomenon. Caregiver score on CBI was compared with the disease characteristics of PD including duration, severity, and presence of on-off phenomenon. Chi square was applied. P-value of ≤0.05 was taken significant.

## Results

One hundred and fifty-six patients diagnosed with PD for more than one year were included in the study from the outpatient PD clinic. Parallel to this group, 156 caregivers were also included. There were 107 (68.5%) males and 49 (31.4%) females in the PD group. Their mean age was 63.14 ± 7.52 years. There were 112 (71.8%) women and 44 (28.2%) men in the caregiver group. Their mean age was 47.75 ± 11.98 years.

When severity of PD was assessed, it was found to be comparable in the study sample. There were 25 (16.0%) patients with Stage 1 PD, 29 (18.5%) with Stage 2 and 3 each, 37 (23.7%) with Stage 4 PD, and 36 (23.0%) with Stage 5 PD. There were 70 patients (44.8%) who were living with PD for less than 10 years and 86 patients (55.1%) who were living with PD for more than 10 years. On-off phenomenon was present in 59 patients (37.8%) with PD.

Overall there were 98 (62.8%) stressed caregivers and 58 (37.2%) non-stressed caregivers as assessed by CBI. The correlation of caregiver stress with the characteristics of the PD patients including severity of disease, duration of disease, and presence of on-off phenomenon is shown in Table 1.

**Table 1:** Caregiver stress and disease characteristics of patients with Parkinson’s. PD: Parkinson's Disease; HY Scale: Hoehn and Yahr Scale; CBI: Caregiver Burden Inventory Stressed: CBI score ≥36 Not stressed: CBI score <36

Characteristics of the PD patients	CBI category for caregivers		P-value
	Stressed n (%)	Not stressed n (%)	
Severity of PD on HY scale			
Stage 5	29 (80.5%)	7 (19.5%)	0.0008
Stage 4	25 (67.5%)	12 (32.5%)	
Stage 3	20 (69.0%)	9 (31.0%)	
Stage 2	17 (58.6%)	12 (41.3%)	
Stage 1	7 (28.0%)	18 (72.0%)	
Duration of diagnosis			
Less than 10 years	29 (41.5%)	41 (58.5%)	<0.00001
More than 10 years	69 (80.2%)	17 (19.7%)	
On-off phenomenon			
Present	48 (81.3%)	11 (18.6%)	0.002
Absent	50 (51.4%)	47 (48.5%)	

As seen in Table 1, 28% caregivers of PD persons in stage 1 were stressed on CBI, but 67.5% and 80.5% caregivers of PD patients in stage 4 and 5 respectively were stressed. Similarly, caregiver stress was significantly more severe in persons diagnosed with PD for more than 10 years (p < 0.00001) and in those with the on-off phenomenon due to levodopa use (p = 0.002).

The caregiver stress level was also correlated with their own gender. Among the stressed caregivers, 85 out of 98 were women (86.7%) and only 13 (13.3%) were men (p-value < 0.0001).

## Discussion

Chronic diseases, especially psychiatric and neurological with progressive course, have long-term debilitating effects on the psychosocial health not only on the patients themselves but also their caregivers. The outcomes of this study signify that more than half of the caregivers of Parkinson’s patients were stressed. Their stress was related to the longer duration of PD, higher severity of PD, and the presence of on-off phenomenon in their patients.

With an estimate of 450,000 individuals affected with Parkinson’s disease in Pakistan, we could not find any study that assessed the psychological health of Parkinson’s caregivers. Hence, to our best knowledge, this is a first of its kind data being reported about the mental health of PD caregivers in Pakistan. However, this is also a small study with its limitations. It was based in one institution only and cannot be generalized to the population. Only one parameter of mental health - stress - was assessed. The bias of other reasons of stress cannot be excluded.

Caregivers of persons with Parkinson’s have been recognized to have poorer quality of life, less social activities such as spending time outside the house or taking vacations. Especially with caregiver spouses, the problem of age-related hindrances is severe. They ever fear falling sick [13]. In a Spanish study, 60% of PD caregivers were afflicted with moderate to severe stress. They reported agitation, hallucinations, lability in persons with PD to be the predisposing factors [14]. Caregivers of PD patients in an Indian study revealed stress of finances, frustration with slow improvement in PD, mental and physical exhaustion, and anxiety and depression [15]. As finances have posed as a worry to Indian PD caregivers, it must be a stress trigger in Pakistani PD caregivers too. India and Pakistan share family structure where mostly there is one or more earning members for a large joint family. There is also little concept of healthcare insurance in this part of the world. This aspect of caregiver stress must be further studied in Pakistani population.

As the disease progresses with time, the severity of psychological symptoms also becomes more intense in caregivers. This finding is reinforced in this study too. It was also seen that caregivers of PD patients without cognitive impairment and dementia were significantly less stressed as compared to those caregivers of PD patients who had longer disease duration and more complications including cognitive impairment and dementia [6].

The overall incidence of psychological symptoms is high in caregivers of PD patients. As the disease progresses and functional and cognitive impairment ensues, the symptoms worsen. Robust studies to assess the psychosocial health of PD caregivers must be conducted in Pakistan. Caregiver health must be evaluated for all relevant psychological parameters including stress, anxiety, depression, fatigue, burnout, social isolation, and overall quality of life. Caregiver health must be evaluated cyclically. Caregivers with deteriorating psychological health, once identified, must be provided counselling and therapy to improve their quality of life.

## Conclusions

Psychological health of caregivers of persons with Parkinson’s is bleak. As the disease progresses, they further succumb to debilitating stress and depression. There is a dearth of literature to extensively comprehend their mental state over time. Qualitative and quantitative studies must be conducted in Pakistan to understand the psychosocial status of PD caregivers and plan strategies to improve their quality of life.
